# EURO-NMD registry: federated FAIR infrastructure, innovative technologies and concepts of a patient-centred registry for rare neuromuscular disorders

**DOI:** 10.1186/s13023-024-03059-3

**Published:** 2024-02-14

**Authors:** Antonio Atalaia, Dagmar Wandrei, Nawel Lalout, Rachel Thompson, Adrian Tassoni, Peter A. C. ’t Hoen, Dimitrios Athanasiou, Suzie-Ann Baker, Paraskevi Sakellariou, Georgios Paliouras, Carla D’Angelo, Rita Horvath, Michelangelo Mancuso, Nadine van der Beek, Cornelia Kornblum, Janbernd Kirschner, Davide Pareyson, Guillaume Bassez, Laura Blacas, Maxime Jacoupy, Catherine Eng, François Lamy, Jean-Philippe Plançon, Jana Haberlova, Esther Brusse, Janneke G. J. Hoeijmakers, Marianne de Visser, Kristl G. Claeys, Carmen Paradas, Antonio Toscano, Vincenzo Silani, Melinda Gyenge, Evy Reviers, Dalil Hamroun, Elisabeth Vroom, Mark D. Wilkinson, Hanns Lochmuller, Teresinha Evangelista

**Affiliations:** 1 Inserm Center of Research in Myology, Neuro-Myology Service G.H. Pitié-Salpêtrière, Sorbonne Université, Paris, France; 2 Clinical Trials Unit, Medical Center – University of Freiburg, Faculty of Medicine, University of Freiburg, Freiburg, Germany; 3 Medical BioSciences Department, Radboud University Medical Center, Nijmegen, Netherlands; 4 Duchenne Parent Project, Veenendaal, The Netherlands; 5 Children’s Hospital of Eastern Ontario Research Institute, Ottawa, Canada; 6 World Duchenne Organisation, Veenendaal, The Netherlands; 7 Duchenne Data Foundation, Bergen Op Zoom, The Netherlands; 8 European Reference Network for Rare Neuromuscular Diseases EURO-NMD, Institute of Myology, University Hospital Pitie-Salpetriere-APHP, Paris, France; 9 Department of Clinical Neurosciences, University of Cambridge, Cambridge, UK; 10 Department of Clinical and Experimental Medicine, Neurological Institute, University of Pisa, Pisa, Italy; 11 Department of Neurology/Center for Lysosomal and Metabolic Diseases, Erasmus MC University Medical Center, Rotterdam, The Netherlands; 12 Department of Neurology, Neuromuscular Diseases Section, University Hospital Bonn, Bonn, Germany; 13 Department of Neuropediatrics and Muscle Disorders, Faculty of Medicine, Medical Center – University of Freiburg, Freiburg, Germany; 14 Unit of Rare Neurological Diseases. Department of Clinical Neurosciences, Fondazione IRCCS Istituto Neurologico Carlo Besta, Milan, Italy; 15 Neuromuscular Diseases Reference Center, Pitié-Salpêtrière University Hospital, APHP Paris, Paris, France; 16 Association Institute of Myology, Hôpital Pitié-Salpêtrière, Paris, France; 17 Association Française Contre Les Myopathies, AFM-Téléthon, Evry, France; 18 European Patient Organisation for Dysimmune and Inflammatory Neuropathies, Paris, France; 19 Neuromuscular Center, University Hospital Motol, Prague, Czech Republic; 20 Department of Neurology, Maastricht University Medical Center+, and MHeNS, School for Mental Health and Neuroscience, Maastricht University, Maastricht, The Netherlands; 21 Department of Neurology, Amsterdam University Medical Center, Location Academic Medical Center, Amsterdam, The Netherlands; 22 Department of Neurology, University Hospitals Leuven, and Laboratory for Muscle Diseases and Neuropathies, Department of Neurosciences, KU Leuven, and Leuven Brain Institute (LBI), Louvain, Belgium; 23 Hospital Universitario Virgen del Rocío/IBiS, Avda Manuel Siurot S/N, 41013 Seville, Andalucía Spain; 24 Department of Clinical and Experimental Medicine, AOU G. Martino Di Messina, University of Messina, Messina, Italy; 25 Department of Neurology and Laboratory of Neuroscience, IRCCS Istituto Auxologico Italiano, Milan, Italy; 26 ALS Liga Belgium, Louvain, Belgium; 27 CHRU de Montpellier, Direction de la Recherche et de L’Innovation, Hôpital La Colombière, Montpellier, France; 28 Departamento de Biotecnología-Biología Vegetal, Escuela Técnica Superior de Ingeniería Agronómica, Alimentaria y de Biosistemas, Centro de Biotecnología y Genómica de Plantas UPM-INIA, Universidad Politécnica de Madrid (UPM), Instituto Nacional de Investigación y Tecnología Agraria y Alimentaria (INIA/CSIC), 28223 Madrid, ES Spain; 29 Neuromuscular Pathology Functional Unit; Neuropathology Service, Institute of Myology, University Hospital Pitié-Salpêtrière-APHP, Paris, France

**Keywords:** Registry, Registry Hub, Neuromuscular Diseases, Rare Diseases, FAIR data

## Abstract

**Background:**

The EURO-NMD Registry collects data from all neuromuscular patients seen at EURO-NMD's expert centres. In-kind contributions from three patient organisations have ensured that the registry is patient-centred, meaningful, and impactful. The consenting process covers other uses, such as research, cohort finding and trial readiness.

**Results:**

The registry has three-layered datasets, with European Commission-mandated data elements (EU-CDEs), a set of cross-neuromuscular data elements (NMD-CDEs) and a dataset of disease-specific data elements that function modularly (DS-DEs). The registry captures clinical, neuromuscular imaging, neuromuscular histopathology, biological and genetic data and patient-reported outcomes in a computer-interpretable format using selected ontologies and classifications. The EURO-NMD registry is connected to the EURO-NMD Registry Hub through an interoperability layer. The Hub provides an entry point to other neuromuscular registries that follow the FAIR data stewardship principles and enable GDPR-compliant information exchange. Four national or disease-specific patient registries are interoperable with the EURO-NMD Registry, allowing for federated analysis across these different resources.

**Conclusions:**

Collectively, the Registry Hub brings together data that are currently siloed and fragmented to improve healthcare and advance research for neuromuscular diseases.

**Supplementary Information:**

The online version contains supplementary material available at 10.1186/s13023-024-03059-3.

## Background

Rare diseases (RD) often remain undiagnosed or misdiagnosed for years, the knowledge about many conditions continues to be sparse [1], and effective, disease-modifying therapies are only available for a minority of RD patients [2]. The challenges for research are multiple: patient populations are typically small, heterogeneous, and geographically scattered, and patient data are scarce and fragmented leading to insufficient knowledge about the epidemiology, natural history and pathophysiology of most conditions. European and international collaboration has long been recognised as the most appropriate way to tackle RD-specific barriers [3, 4]. The European Reference Networks (ERNs,https://health.ec.europa.eu/european-reference-networks), working closely with patient organisations, have triggered cross-border EU collaboration between healthcare providers (HCPs) for complex and rare diseases [5]. One of the axes of this collaboration is the development of Registries to measure and guarantee a homogeneous delivery of care across the European space, the identification of cohorts and populations, enhancing clinical trials recruitment, collecting neuromuscular diseases long-term data and promoting research [4].

Consequently, the EU Health Programme has allocated funds so ERNs can develop registries with mixed clinical monitoring and research purposes. The use of registries allows data pooling for adequately-powered statistical analysis, thereby overcoming rare diseases' knowledge gap in epidemiological and clinical research [6–8]. However, difficulties still emerge from duplication of efforts and lost opportunities arising from the diversity of registries covering similar areas, data quality issues, proprietary formats and reduced interoperability.

## Main Text

EURO-NMD is the European Reference Network for Rare Neuromuscular Diseases, collectively affecting 500,000 EU citizens [9]. The number of patient registries for neuromuscular disorders in Europe and worldwide is estimated to be several hundred. These registries may have been established at a centre, regional, national or international level, often dedicated to a single neuromuscular condition and a specific purpose [10–12]. They may use patient-reported data, clinician-reported data or a combination of the two, and the number of data elements collected may range from less than ten to several hundred. The choice of data elements is primarily determined by the purpose of the registry, as a registry aiming to assist in the recruitment of participants into clinical trials may collect a different set of data to one seeking to diagnose individuals who lack a molecular diagnosis or to collect longitudinal clinical data on a patient cohort. In recent years, efforts have been made to harmonise data elements across different registries, particularly those related to the same condition [13, 14]. Ontologies help make data more machine-readable and interoperable [15]. The use of the Human Phenotype Ontology (HPO), particularly, was found to facilitate diagnostic research and gene discovery for rare diseases, including neuromuscular diseases [16]. With the recent marketing approval of often costly disease-modifying treatments in several neuromuscular disorders, e.g., Spinal Muscular Atrophy, additional data collection that better captures clinical progression and response to treatment has been initiated [17]. Data captured in post-marketing registries to generate real-world data may include specific functional, laboratory or imaging tests and patient-reported outcomes [18].

## The EURO-NMD registry hub

### EURO-NMD registry hub responds to clinicians’ and patients’ unmet needs

Neuromuscular disorders are challenging to recognise, and patients experience long diagnosis delays, a phenomenon coined the “diagnostic odyssey” [19]. Few specific treatments have been described, as they cover less than 5% of rare diseases, but their number is multiplying for gene and variant-specific therapies [20]. EURO-NMD is one the largest ERNs bringing together 82 healthcare providers across 25 European countries and including 27 patient organisations. The Network works to speed up diagnosis and research in NMDs and improve the standards of care for these pathologies. It is structured around five main disease groups: Muscle Diseases, Peripheral Nerve Diseases, Neuromuscular Junction Defects, Mitochondrial Diseases and Motor Neuron Diseases (https://ern-euro-nmd.eu/).

**Fig. 1:**
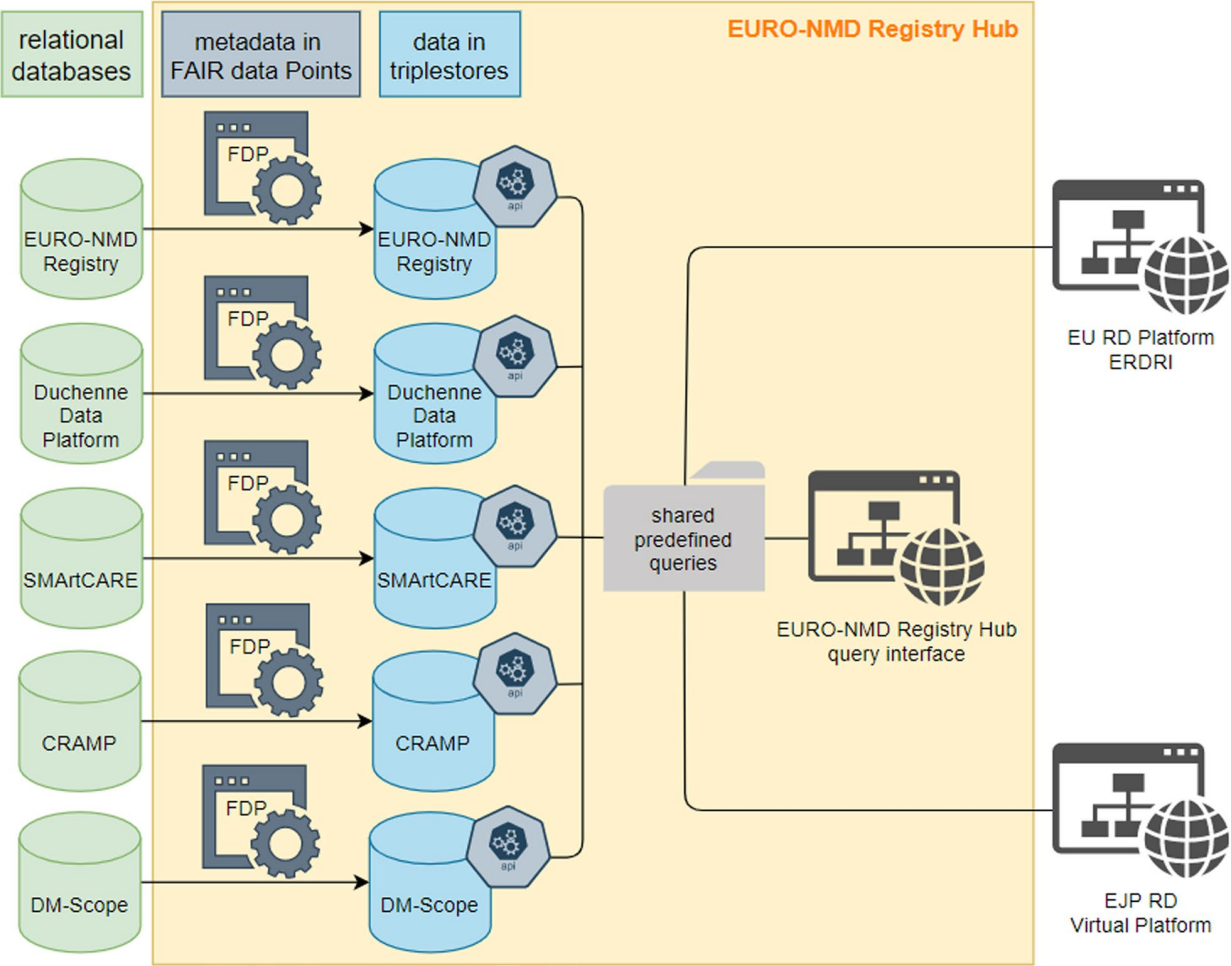
EURO-NMD registry hub

More than 100,000 NMD patients are estimated to be seen annually by the HCPs in the ERN. EURO- NMD health care providers and patient organisations are active in more than 120 primarily disease-specific and patient-run registries. While the existing registries collect vital information, none is used by all EURO-NMD centres, and there is no unified NMD or NMD Disease-Specific Registry in the EU. Data from the EURO-NMD registry was set up to be interoperable with four existing pilot registries (Duchenne Data Platform, CRAMP, DM-SCope and SMArtCARE) as a proof-of-concept of the EURO-NMD Registry Hub framework (Fig. 1).

The EURO-NMD Registry Hub project (https://registry.ern-euro-nmd.eu/) was launched in May 2020 with equal co-funding from the EU 3rd Health Programme and three leading patient organisations, World Duchenne Organisation, Duchenne Data Foundation and AFM-Téléthon (Grant agreement 947598). The objective is to build a patient-centred and interoperable registry hub for all paediatric and adult patients with rare neuromuscular diseases treated within the EURO-NMD's Centres, including undiagnosed patients. It was designed to collect longitudinal clinical and patient-reported data and exchange data with existing registries by adopting FAIR data management and stewardship principles and EU standards for data collection [21]. The EURO-NMD registry is a component of the Hub that addresses the data collection needs of the healthcare providers that are members of the ERN.

### Importance of patient involvement and in-kind contribution

The EURO-NMD Registry Hub is designed as a clinician-patient partnership. Even if it primarily aims to collect clinical data from patients seen at EURO-NMD's expert centres, it addresses patients' unmet needs. The consenting process covers uses beyond the clinical ones, such as research, cohort finding and trial readiness when patients agree to be contacted to re-consent for those specific uses. Patients have a say at all levels of the registry life and input medical-grade patient-originated data in the form of patient-reported outcomes, adding an information layer for long-term monitoring of diseases and treatments.

The EURO-NMD Registry Hub project has been supported by three patient organisations, AFM-Téléthon, Duchenne Data Foundation, and World Duchenne Organisation, who recognize the significance of connecting neuromuscular data to improve health outcomes. These organisations have provided a shared in-kind contribution of 274,641.20€, which supplements the project's budget and enables the registry to benefit from the expertise and resources of patient organisations.

In-kind contributions from patient organisations in research projects are essential for several reasons:

1. *Expertise*: Patient organisations often have extensive knowledge and experience in the specific disease or condition being researched, as well as in the needs and priorities of the patient community. Their expertise can be valuable in shaping the research agenda and ensuring patient-centred research.
2. *Patient involvement*: In-kind contributions from patient organisations can facilitate patient involvement in the research process, from study design to dissemination of results. This involvement can help ensure that research is meaningful and relevant to patients and increase patient trust and participation in the research process.
3. *Resource leveraging*: In-kind contributions from patient organisations can help leverage resources and maximize the impact of funding. Patient organisations can contribute staff time, expertise, and other resources that can supplement the research project's budget and increase the project's overall impact.
4. *Collaboration*: In-kind contributions from patient organisations can facilitate cooperation between patients, researchers, and other stakeholders. This collaboration can foster a shared understanding of research goals and priorities and help build relationships leading to future collaborations.
5. *Co-governance*: Patient organisations have the same rights and obligations as all other partners in the project and are equal partners in the research process. Co-governance can help ensure that the perspectives and priorities of patients are integrated into all aspects of the research project, from design to dissemination of results.

Overall, in-kind contributions from patient organisations can help ensure that research is patient-centred, meaningful, and impactful and can facilitate collaboration and resource leveraging to maximize the impact of research funding. Co-governance can help build trust and foster a sense of ownership among patient organisations, researchers, and other stakeholders, ultimately leading to more meaningful and impactful research outcomes.

## EURO-NMD registry

A primary objective of the Registry was implementing a continuous monitoring system to track EURO-NMD's Centres' performance against selected key performance indicators (KPIs) of care quality while providing sufficient data to launch research and clinical trials and inform policy and regulatory decisions.

### Registry ethics, privacy and security

The EURO-NMD Registry is housed within the secure hospital IT infrastructure of the University Medical Centre Freiburg in Germany. All data is stored within this secure infrastructure and can only be accessed by authorised personnel. Web access is only possible for authorised users with verified credentials over a secure channel with encryption (HTTPS). Backups are performed regularly, and a disaster recovery procedure is in place.

The Registry uses an opt-in approach for recruitment, meaning that hospitals must obtain informed consent from patients for participation. Ethics and governance approval are required before each hospital participates in the EURO-NMD Registry, and each centre has a Data Protection Impact Assessment (DPIA) in place. Pseudonymisation is also implemented, with all patients assigned a pseudonym by the site entering the data. The patient's identity is only known by the hospital responsible for the patient care. Data entered by a participating hospital is not shared with other users except in aggregated format for benchmarking. Data are available in aggregated and/or pseudonymised formats to all stakeholders to develop projects, policies or studies, following approval by the Data Access Committee of the EURO-NMD Registry Hub project, which includes HCPs and patient representatives. Patients registered to the EURO-NMD Registry can also access and manage their own clinical and patient-generated data. Stakeholders beyond the Reference centres within EURO-NMD include other ERNs, investigators from outside the ERN, patient organisations and patient groups, government organisations, not-for-profit organisations, and potentially industry and pharmaceutical companies. However, there is no current framework for these interactions yet in place. Different access levels are defined depending on the stakeholder group applying to use the Registry data.

### Patients’ role in governance and data access

Based on an extensive qualitative survey conducted by EURORDIS in 2019, most rare disease patients want to share their health data to advance scientific research and clinical benefits but simultaneously express specific preferences, needs, and concerns regarding data sharing. The results demonstrate specifically the willingness of almost half of the survey respondents (54%) to have more control over their private health data and the data sharing process [22].

One of the innovative components of the EURO-NMD Registry functionality is that the patient is the ultimate manager of his data. The three patient advocacy groups, as equal partners in the co-design of the Registry, have been involved in the discussions related to common standards for data privacy and security, FAIRification of data, storage, access, and data curation (e.g. data organisation/management, quality assurance) while ensuring full compliance with GDPR and ethical principles. It is also imperative at this stage to explore the future development and implementation of dynamic systems tailored to the needs and preferences of patients, regarding whom they share their data, for which purpose and flexibility to change their preferences reliably and at any time. Also, patients’ access to updated information on research outcomes to which their data has contributed will foster patient participation in data-sharing initiatives [6].

Role-based rules have been defined for the different types of stakeholders for the data access and analytical purposes of the EURO-NMD Registry, as described in detail in the data access policy manual (publicly available at the registry website https://registry.ern-euro-nmd.eu).

In the first phase, only patients followed by specialists from the ERN EURO-NMD Full Members or Affiliated Partners will have access to the EURO-NMD Registry. Patients can have access to their data, and they will be able to complete online patient-reported outcomes/surveys. Furthermore, we envision a dynamic, machine-assisted consent process for the patients to grant each potential user of their health data a nonexclusive license to use such data for research purposes.

### Registry IT architecture

Data from the EURO-NMD Registry will be collected and managed using Research Electronic Data Capture (REDCap) software hosted at University Medical Center Freiburg [10, 11]. The objective is to design an interoperable tool to gather one-entry epidemiological data with dynamic, recurrent data collection to reflect disease progression and treatment effects. In the latter case, both scheduled and unscheduled annotations should be possible. Another central requirement for establishing the EURO-NMD Registry was to define five disease-driven datasets (Neuropathies, Myopathies, Mitochondrial Diseases, Neuromuscular Junction Disorders, and Motor Neuron Diseases) and a dataset for undiagnosed patients. These six subsets represent a “branch” of the Redcap data collection. During data entry, patients are assigned to one of those branches. The instruments (i.e., individual forms) and data items shown are customised based on the choice of dataset during data entry, facilitating data entry and saving time spent on that task.

The logic of the data organisation goes beyond disease groups, as there are other criteria on which branching and the inclusion or exclusion of specific data items and instruments depend, such as age, gender, clinical diagnosis or the affected gene in the case of a mutation.

The temporal nature of the longitudinal data led to the definition of three time points:

- "Enrolment": for all information to be collected once (e.g., demographics, consent)
- "Visit" for all information that is updated at least annually and can typically be determined during a patient encounter (e.g., phenotypes, all longitudinal data to enable natural history assessment)
- "Unscheduled" for all information that should be continuously traceable and does not necessarily coincide with a scheduled patient encounter at the specialised HCP / ERN centre (e.g., changes in medication, results of genetic tests).

Several validation mechanisms have been implemented to ensure quality while entering the data.

Firstly, there are default data types for all items, predefined response options and distinguishability between "not answered" and "unknown." The entry of free text is avoided.

Secondly, data quality rules are in place that prevent implausible entries (e.g., the visit data cannot precede the date of the consent). On the other hand, whenever possible, the entries are linked to ontology search and validation. For example, registry users can enter the name of a disease, and the system will return a list of codes from the Orphanet Rare Disease Ontology (ORDO) for selection (available at https://www.orphadata.com/ordo/).

Finally, an audit trail tracks which user changed a data item and when and which data exports were called up.

Users can view summary statistics for their centre's patients via project dashboards and download predefined data exports in different formats (e.g., CSV, SPSS, SAS, R, Stata, CDISC).

The technical requirements for the system were formulated as 24 technical Key Performance Indicators (technical KPIs), available as Additional file 2. They include, for example: "secure data transmission/storage ", "role-based access ", "follow FAIR Principles ", "track changes ", "data export ", and a more detailed description for each requirement. We have formulated one or more test cases with step-by-step instructions and expected results for each technical KPI. Based on these operationalised requirements, the system was tested by one of the patient organisations involved in the project with experience in digital application development and data management (Data Duchenne Foundation). All tests achieved the desired results. A public report on the data audit related to the data quality and completeness is available.

We nominated a group of twenty test users among all disease group representatives from many different countries to evaluate the software's usability and adequacy through mock data entry. The assessment of the users' satisfaction with the implementation was positive. We organised a hands-on session explaining the basic features of the software before starting the feedback collection. We have recorded this session and set up a quick-start guide to assist the testers. We asked the latter to enter patient data into the system as close to reality as possible while changing all identifiable information. We provided a survey for the structured collection of feedback and feature requests and also asked the testers to report their overall impression in a partially standardised "User experience"-survey.

As a result of the received feedback, we have modified the tools for genetic diagnosis to allow, for instance, for better mapping of variants of mitochondrial DNA and a more accessible listing of previously performed tests that did not return diagnostic results.

The conclusions of the user-experience survey so far indicate that the system is understandable, works reliably and that the collected data is considered relevant for patient care and research of neuromuscular diseases. The feedback on time needed for data entry and the completeness of the collected data items and response options was comparatively heterogeneous and clearly illustrates a typical conflict of objectives.

### Registry site operations

The HCPs members of the network are requested to enroll all neuromuscular patients seen at the centre, both prevalent and incident. As a rule, each patient should have at least an annual registry data entry.

The EURO-NMD Registry uses a hybrid registry model that fully complies with the European General Data Protection Regulation (GDPR) and the national laws and regulations of the different member states. It is a unique set-up that allows HCPs to enter data in a centralised registry or, alternatively, to build local versions of the registry that are in federation with the central registry and each other. The first option means fewer challenges to the local IT infrastructure of the participating HCPs. The second option could offer considerable advantages in overcoming complications by using different protocols and legislation in different countries. Ultimately, the participating institutions will decide on their preferred choice.

### Registry datasets

The Joint Research Centre (JRC) of the European Commission has defined a "Set of common data elements for Rare Diseases Registration". This dataset of 16 common data elements, the EU-CDEs, is freely available on the internet in 21 languages and represents "the first practical instrument released by the EU RD Platform aiming at increasing interoperability of RD registries" [23].

The EU-CDEs have been implemented in all the ERNs registries, enabling cross-talking between them and cross-ERN comparisons. However, EU-CDEs are not disease-specific and may not answer all relevant questions for the EURO-NMD ERN. In addition, while these data elements were mandatory, their implementation in the different registries was not harmonised. As an example, genetic diagnosis is an ERDRI-mandated data item. Still, the only recommendation on the document reads: "International classification of mutations (HGVS) (strongly recommended—see link)/HGNC/OMIM code". Still, annotating the exact genetic defect is essential for many rare diseases. For most ERNs, the level of annotation may vary between disease groups and ERNs, but we adopted a nomenclature compliant with Human Genome Variation Society (HGVS) recommendations.

On top of the EU-CDEs, we have added a Common Neuromuscular Dataset of 44 fields serving all neuromuscular diseases (NMD-CDEs). This dataset is the intersection of the data elements chosen by all five disease areas (muscular diseases, peripheral nerve diseases, mitochondrial diseases, neuromuscular junction diseases and motor neuron diseases). It applies to any neuromuscular patient seen in a node of the network. Finally, every disease area has Disease-Specific Datasets (DS-DEs). These have a reduced number of fields, only displayed if the diagnosis points towards their inclusion in the visit data collection form.

The crucial starting point for the EURO-NMD registry was to select the data elements that serve its primary purpose. All data elements were reviewed according to the following priming questions:

- Does the data element contribute to the primary mission of EURO-NMD, i.e., improving healthcare for rare neuromuscular conditions across the HCPs?
- Is the data element required at the time of diagnosis (baseline) or subsequent visits (follow-up)?
- Is the data element required across all age groups, or only in children or adults?
- Is the data element required for all neuromuscular diseases, or just for one or several of the disease groups (e.g., myopathies), or just for a specific disease entity (e.g., Duchenne muscular dystrophy)?

**Fig. 2:**
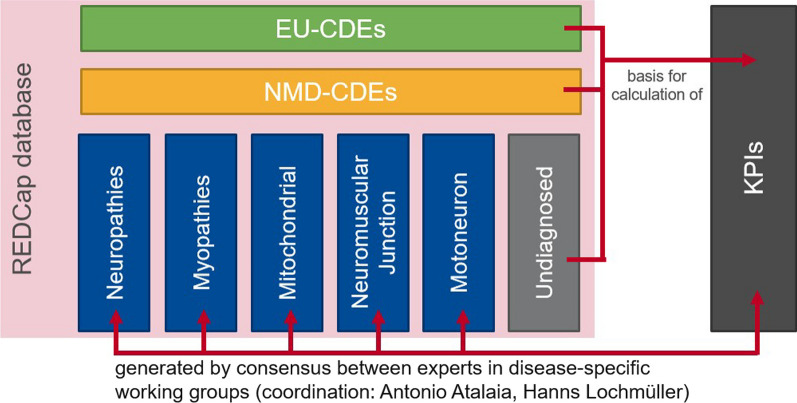
Sets of data items in the EURO-NMD registry

Figure 2 visualises the composition of the registry from group-specific datasets and the intersections across groups resulting from NMD-CDEs and EU-CDEs.

All data elements and their hierarchical dependencies are described in the codebook and adopt standard ontologies as recommended for data integration [24]. We do not exclude future inclusion of different or additional ontologies when needed. However, the current choice, depicted in Table 1, reflects the experience of existing neuromuscular registries. The codebook is available to the registry users via the REDCap platform.

**Table 1:** EURO-NMD registry hub adopted ontologies

Ontology	Use	Website
Human phenotype ontology	Semiology definitions	https://hpo.jax.org/app/
Human genome variation society	Annotation of modification at chromosomal, gene, variant and protein level	https://www.hgvs.org/
OMIM	Classification of genes and diseases	https://www.omim.org
ORDO (orphanet rare disease ontology)	Classification of rare diseases	https://www.orphadata.com/ordo/

### FAIR-by-design

FAIR stands for Findable, Accessible, Interoperable, and Reusable. It is the acronym used to describe a global initiative to make data more valuable by increasing the ability of computers to find, interpret, integrate, and analyse those data autonomously.

Findable means that healthcare professionals, researchers, regulators, representatives of patient organisations can find EURO-NMD registry and see what data exist. This is achieved through registering the EURO-NMD in JRC’s ERDRI platform (https://eu-rd-platform.jrc.ec.europa.eu/erdridor/register/6501). Accessible means that once they find the data they were looking for, they clearly know if they are authorised to use them. Interoperable means that records written in different languages and formats can be combined and Reusable means that data can be used more than once for research but also by patients themselves.

The EURO-NMD Registry will be interoperable with other rare disease registries under a data-sharing model relying on federated queries that run without data transfer between registries ('the data visiting' approach). In this light, we designed and integrated a FAIR architecture (FAIR-by-design) that complements existing ERN EURO-NMD data management systems. This design addresses two critical concerns related to the sharing of patient health data: protection and privacy. The FAIR infrastructure implements data access regulations by preparing patient health data for secondary use in a secure (anonymized and aggregated data) and GDPR-compliant way.

Interoperability is achieved by processing a subset of the data collected from each participating Registry according to the semantic model for the EU-CDEs developed by EJPRD (European Joint Programme on Rare Diseases) and hosting this transformed data in a triplestore, which is a component of a FAIR Data Point. A FAIR Data Point (FDP) is "a metadata repository specifically designed to adhere to as many FAIR Principles and norms as possible. It follows a global standard for descriptions of catalogues and datasets (Data Catalog Vocabulary—DCAT)) and enhances them by adding the fully machine-actionable read/write behaviours of the Linked Data Platform, allowing machines to explore the data descriptions autonomously. FDPs also automatically register themselves in a central index, which browses their metadata regularly, thus enabling "smart search" overall public FDPs making this software an indispensable component of a FAIRification process. Apart from being a software that allows data owners to expose datasets in a FAIR manner and data users to discover information about the offered datasets, it acts as a gateway to data by describing the access conditions and any restrictions enforced by the repository.

A FAIR assessment of the newly-built EURO-NMD registry was achieved using an automated evaluation system called the 'FAIR Evaluator', developed as an open source (https://w3id.org/AmIFAIR). The EURO-NMD registry scored 19 out of 22 possible points, representing 22 individual tests spanning all FAIR principles except for R1.3 (The (meta)data conforms to community standards). The FAIR assessment was particularly useful in guiding the development teams on the quality of their deployment process. It helped detect accidental errors or misunderstandings that were difficult to spot with the human eye. It provided Proof of Compliance with a set of FAIRness requirements supplied by the EURO-NMD Steering Committee through the Registry Key Performance Indicators.

### Registry key performance indicators

To identify data elements contributing to improvements in healthcare, we defined key performance indicators (KPIs) that can be used to benchmark the performance of the HCPs and track outcomes for patients (Additional file 1). Across all neuromuscular diseases, "time to diagnosis" and "time to treatment" were identified as KPIs enabling comparing processes at different HCPs. In KPIs such as "percentage of patients with complete remission" for a particular disease, treatment timeframe allows the comparison of patient health outcomes. Since the KPIs are not directly captured by the registry but are derived indicators that can be generated from data elements at a cohort level, the data elements necessary to derive the KPIs were then defined. For example, "time to diagnosis" can be derived from the date of a patient's first coming to the attention of the expert centre, together with the confirmed molecular diagnosis and the date of the diagnosis.

We invited the five disease group chairs of the ERN EURO-NMD to define key performance indicators (KPIs) and identify the corresponding data items needed for appropriate longitudinal follow-up of the neuromuscular conditions of their expertise. The KPIs are intended to capture standard measurements of HCPs' performance and quality of patient care and inform the participating centres about their respective KPI results.

For example, the KPIs include:

- Average time from the patient's first contact with HCP to diagnosis or start of targeted therapy.
- The proportion of patients with access to therapy services (e.g., physical therapy, occupational therapy, dietary advice).
- The proportion of patients on different types of ventilation.
- The proportion of patients with cardiac disease.

We have defined Key Performance and Outcome Indicators (KPIs) for each dataset that help each HCP range their performance inside the network and guarantee high-quality care delivery to patients. For the Common Data Elements, we intend to collect both KPIs and aggregated statistics results as described below:

- % of cases by age (adult/pediatric)
- % of cases by sex
- % according to patient status
- life expectancy
- % patients diagnosed in the first year
- Time from onset to first HCP visit
- Time from onset to diagnosis
- % clinically diagnosed cases
- % genetically diagnosed cases
- % undiagnosed cases (without clinical/genetics diagnosis)
- % patients consenting to be contacted for research purposes
- % patients consenting to the reuse of their data (by list item)
- % patients with biological samples
- % patients with biological samples in a biobank
- stratification by disability score

The complete list of data elements and corresponding KPIs can be found in Appendix 1.

On every appointment, the collection or updating of the Common Data Elements is mandatory. We encourage a progressive capture of the Common Neuromuscular and Disease-specific Datasets. Still, we will not benchmark the latter during the initial data collection cycle of 2 years.

### Patient-reported outcomes

Patient-reported outcome Measures (PROMs) provide essential information on benefits for patients' health and quality of life and are best captured through standardised questionnaires. Some of these instruments (such as CGI-I, SF36) are generic and not limited to a specific neuromuscular condition, which makes them valuable tools to compare outcomes across many if not all, patients and diseases. However, more granular and targeted outcomes are often required to ascertain the specific health benefits of treatments for specific conditions, so disease-specific outcome measures may also be required to capture treatment-related KPIs for the ERN, which may differ widely when considering a disease such as myasthenia gravis in comparison with a disease such as inclusion body myositis. Together with patient partners in the network, we are currently in the process of identifying a complete list of PROMs to be captured by the EURO-NMD registry. They should ideally be (a) considered relevant by the patient partners themselves, (b) validated for the specific disease and age group, (c) available in multiple European languages, (d) not incur license fees and (e) easy to apply in the waiting room or via the internet from home.

## The EURO-NMD registry hub GDPR-compatible interoperable platform based on FAIR data

FAIR data principles encourage robust management of data and metadata (i.e. data about data) for efficient use and reuse by humans and computers. They are intended to support querying and analysing data stored in different resources to answer specific questions by various stakeholders, such as: “What is the time difference from onset to first HCP visit across Europe?” Querying the EURO-NMD registry will be facilitated by the Virtual Platform developed by the European Joint Programme on Rare Diseases (EJP-RD). The Virtual Platform will offer a graphical user interface (GUI) for humans and an Application Programming Interface (API) for computers to query data across existing registries and the registry hub. However, this can only be possible when the data exposed by various registries are FAIR, where data stay at the source but can be queryable at a distance from an EJP-RD query point (see Fig. 1 for the schematic representation of a privacy-preserving federation over multiple registries).

The FAIR-by-design EURO-NMD registry has built-in interoperability founded on using accepted ontologies and classifications that promote data integration. At the same time, the FAIRification of the datasets is destined to sustain inter-registry interoperability. This was successfully demonstrated in a proof-of-concept for the EU-CDEs, where data from the EURO-NMD registry is now interoperable with four existing pilot registries: Duchenne Data Platform, CRAMP, DM-SCope and SMArtCARE (see Fig. 1). These are examples of one patient-driven, one national, and two clinical registries, respectively. Conducting queries related to neuromuscular diseases is possible without exposing sensitive patient details. The solution is based on technology that connects Web addresses to database queries, thus limiting database exploration to only pre-approved questions (e.g. patient count). The prototype depends on a publicly available database of queries manually curated and filtered by experts in FAIR and neuromuscular diseases. FAIR makes it possible for the same query to be executed over independent resources; thus, sharing those queries leads to convergence between registries.

Queries are executed, on demand, over FAIR datasets through a query interface. All of this is protected by a “proxy”, which further insulates the other components, and ensures only encrypted communication over the Web. Accessing the proxy retrieves, for example, only a count of patients with a given rare disease that can be aggregated in a graphical analytics environment.

### Registry management, stakeholder engagement, sustainability and future-proofing

The EURO-NMD Registry is an essential vector within the European Reference Networks' activities required by the European Commission, which funds and oversees the different network activities. For the Commission, the registry's essential feature is monitoring the network's clinical activity to guarantee homogeneous care delivery across the European space, thus demonstrating the added value of the ERNs approach for Rare Diseases management. Furthermore, the registry supports research, regulatory activities and the demand for therapies for rare neuromuscular diseases. Critical mass generation through the registry datasets and identification of cohorts that ensure rare diseases trial-readiness are evident benefits of the platform. Aspects of natural history and long-term monitoring of conditions and treatments concomitantly serve regulatory and policy decisions.

The guiding principles of the registry guarantee that its usefulness matches its legal and ethical high standards. We have adopted previously published eight principles [25]:

1. Transparency.
2. Accountability.
3. Follow the rule of law.
4. Integrity.
5. Participation and inclusiveness.
6. Impartiality and independence.
7. Effectiveness, efficiency and responsiveness.
8. Reflexivity and continuous quality improvement.

The first five points are sufficiently documented in this paper. The guarantee of impartiality and independence calls for an external body to evaluate our procedures, and it is currently performed by the European Commission and the monitoring mechanisms it has put in place. The need for a future body in the form of an External Auditor Body is a discussion still being processed. We have simultaneously put in place Key Performance Indicators for clinical outcomes and processual ones that will help comply with requirements of effectiveness, efficiency and responsiveness. As we currently start using the registry, our primary focus is on reflexivity and continuous quality improvement, and we trust our members to play an active role in these tasks. We intend to designate a registry champion per HCP, to liaise locally and incentivise the registry use. Despite our limited budget, we need to work on variables of staffing and funding. We intend to reward the best performing centres and actively support the ones with the most difficulties.

The registry aims to protect the privacy and safety of patients while allowing the correct use of the collected data. An all-stakeholders Data Access Committee will care for the transparency and legitimacy of data use through a strict role-based access system. The Data Access Committee comprises the Registry Coordinator, Scientific Project coordinator, FAIR data expert, IT specialist, Project facilitator and two patient representatives. The DAC members serve for 3-year terms with an option of extension. The DAC receives and evaluates the requests for data access, except for the public data counts on the website.

The responsibilities of the DAC are:

- Treat all data requests confidentially.
- Aim to respond promptly to all data requests and provide adequate feedback.
- Check that the proposed work complies with the terms and conditions of the ethics approval provided to the EURO-NMD registry.
- Look for evidence that the third-party requesting data is appropriately qualified for the use of the data.
- Advise on project overlaps or improvements that will optimise data use/reuse.
- Be aware of their conflicts of interest.
- Ensure that the effort of all those involved is appropriately acknowledged.

There will be discovery data publicly available on the website consisting of counts centred on the Common Data Elements, which will have some delay to the live database. The access to data varies according to the stakeholders involved, and some access levels are only allowed after the Data Access Committee review.

The data access roles we have defined include:

- Contributing researcher
- Non-contributing researcher
- National Health Authority
- Regulatory Authority or Health Technology Assessment (HTA) entity
- Insurance Companies/Payers
- Non-Governmental Organisation (NGO)
- Patient Organisation
- Individual Patient

Individual patients will have permanent access to their data immediately after collection, and patient organisations will have differentiated access to data according to the involvement or not of the DAC.

Other roles have limitations regarding access to data; some of it is available for discovery, but most of the data is governed by the Data Access Committee (from counts to pseudonymised data, CDEs-only to all data) according to defined criteria by the Registry Steering Committee.

Finally, the EURO-NMD Registry Hub will connect the EURO-NMD Registry with other data sources in an innovative IT platform solution that will significantly benefit patients, their families and patient organisations by developing a linked system that will enable anonymised health data to be accessed and interlinked through multiple existing data sources across different EU countries. The EURO-NMD Registry Hub is expected to be linked to patient community websites and promote the publication of relevant information to the patient organisations and patients. The sustainability of the EURO-NMD Registry Hub is connected with the engagement of the different project partners (Academia, Care Centres, and Patient Organisations) through communication and dissemination activities and participation of the Registry Hub in EU funding programmes, as well as feasibility and research studies promoted by the industry or academics.

The EURO-NMD registry and EURO-NMD registry hub bring innovation, as we wanted to avoid building another clinicians-only-based registry that would not serve also the unmet needs of patients. Another characteristic we underline is that patients are positioned as the managers and have full access to their data to assist with their care or consent to participation in research. The data in our platform never exits its original location, as the federated queries run on distributed agents containing just enough aggregated or anonymised data to answer the questions. In circumstances that the Data Access Committee deems adequate, the re-identification of patient cohorts can be done through the pseudo-anonymisation mechanism for the purposes consented to by the patients. Patients' participation in the platform's governance is transversal and guarantees patient oversight of all mechanisms involved. We aim to develop a fully functioning Patient Portal that will guide all interactions with the patient registry and to create a Patient Locker where the patient safely keeps all his disease-related information and decides with whom the data needs to be shared. The latter will be an essential tool for patient empowerment by returning control over their data to patients clearly and quickly.

The Registry Hub for Neuromuscular Diseases projects itself as a multi-stakeholder tool essential for diagnostic innovation and treatment discovery. It is evolving from an EU-funded project to a multi-national-based healthcare and research tool able to monetize deliveries for commercial purposes while providing resources for tasks such as HTA, integral for regulatory and policy purposes allocated to existing financial streams. We envision this mixed model as the source of the platform's sustainability and are committed to the continuity and improvement of its architecture and workflows in the long term. With that in mind, we work on an API and a sharing agreement to enable other institutions starting a registry to use the same codebook as our platform and optimise the interoperability and reuse of data collected via the new register.

## Discussion

This paper presents an innovative solution for building a Registry platform for the European Reference Network for Rare Neuromuscular Diseases. Since hundreds of registries for neuromuscular diseases already exist, the concept of the EURO-NMD Registry Hub is to reuse existing data instead of duplicating existing data sources with yet another siloed data collection. One strong argument in favor of this sharing model is that data participating in the Hub never exits its original location, as the federated queries run on distributed agents containing just enough aggregated or anonymised data to answer the questions.

Our tested interoperability with four existing registries (the Duchenne Data Platform, CRAMP, DM-Scope and SMArtCare) is a significant milestone. It is now possible to conduct the same queries related to neuromuscular diseases in multiple independent registries simultaneously without exposing sensitive patient details. Whenever possible, and to reinforce alignment of efforts and sustainability, we will work on creating FAIR Data Points for any platform. By sharing the same ontologies and classifications, we can return results for any query originating within the ecosystem.

There has been some preliminary work on defining the semantic terms for describing the informed consent processes and contents for data access to and secondary use of patient health data [26]. Machine-readable data use conditions still need to be implemented in the EURO-NMD registry.

We have been working on an API and a sharing agreement to enable other institutions starting a registry to use the same codebook as our platform and optimise the interoperability and reuse of data collected via the new register.

We have invited patients and patient organisations to participate from the inception of the EURO-NMD registry and EURO-NMD registry hub, as we wanted to avoid building another clinicians-only-based registry that would not cater to the unmet needs of patients. Another characteristic that we underline is that patients are positioned as the managers of their data and have full access to their data, either to assist with their care or to allow research to profit from the participation of their data in scientific queries. Patients' participation in the platform's governance is transversal and guarantees patient oversight of all mechanisms involved. We aim to develop a fully functioning Patient Portal that will guide all interactions with the patient registry and to create a Patient Locker where the patient safely keeps all his disease-related information and decides with whom the data needs to be shared.

This will be an essential tool for patient empowerment by returning control over their data to patients clearly and quickly.

## Conclusions

The EURO-NMD Registry Hub is built to collect and/or connect data from rare neuromuscular patients across different platforms linked through FAIR Data Points, overcoming the siloed data issue that prevents patients from fully benefiting when they contribute their data for care and research. The patient contribution is exceptionally high in domains of governance and data entry of PROs, and also regarding patient access to data and dynamic consent management tools. This creates an information environment that exceeds the usual limits of the registries already in place and empowers patients, while enriching the meaningfulness and validity of the long-term data collected. In conclusion, the FAIR-by-design and strong patient engagement in design and implementation are two innovative pillars of our registry platform. As a result, the registry enables bringing together clinician-entered data, patient reported outcomes and quality of life input.

### Supplementary Information

**Additional file 1:** List of Registry Key Process Indicators (KPIs).

**Additional file 2:** List of database technical Key Process Indicators KPIs.

## Data Availability

Access to data in the registry itself, as well as to the REDCap Codebook listing all data elements captured, is available to authorised users under conditions governed by the registry data access policy as described in this publication and set out on the registry website at https://registry.ern-euro-nmd.eu. The registry KPIs are publicly available as an Annex to this publication. Owing to intellectual property restrictions, the full registry codebook is not publicly available at this time but is available from the corresponding author on reasonable request, subject to certain conditions restricting commercial reuse. The authors highly encourage interested stakeholders to contact the corresponding author for any potential new application of the registry data elements that would support the "reuse" principle of the FAIR data principles.

## Appendix 1: Key process indicators list

**Table:**

*KPI*	KPI's CDEs
% Cases by age (adult/pediatric)	EU-CDEs
% Cases by sex	EU-CDEs
% Patients according to patient status	EU-CDEs
Life expectancy	EU-CDEs
Time from onset to first HCP visit	EU-CDEs
% Patients diagnosed in the first year after first contact with HCP	EU-CDEs
Time from onset (first symptoms) to specific diagnosis	EU-CDEs
% Clinically diagnosed cases	EU-CDEs
% Genetically diagnosed cases	EU-CDEs
% Undiagnosed cases (without clinical/genetics diagnosis)	EU-CDEs
% Patients consenting to be contact for research purposes	EU-CDEs
% Patients consenting reuse of their data (by list item)	EU-CDEs
% Patients with biological samples	EU-CDEs
% Patients with biological samples in a biobank	EU-CDEs
Stratification by WHODAS disability profile/score	EU-CDEs
% Patients with delayed milestones	NMD-CDEs
% Patients with first degree affected relatives	NMD-CDEs
% Patients having received genetic counseling	NMD-CDEs
% Patients by ambulation status	NMD-CDEs
% Patients that lost ambulation in the last 12 months	NMD-CDEs
% Patients with independent feeding capacity	NMD-CDEs
Body height (cm)—percentile	NMD-CDEs
Body weight (kg)—percentile	NMD-CDEs
% Patients with cardiac disease	NMD-CDEs
% Patients with feeding difficulties	NMD-CDEs
% Patients with breathing difficulties	NMD-CDEs
% Patients with cognitive impairment	NMD-CDEs
% Patients with scoliosis	NMD-CDEs
% Patients with acquired cogntive impairment (dementia)	NMD-CDEs
% Patients with development delay	NMD-CDEs
% Respiratory insufficiency	NMD-CDEs
% Patients with symptoms of hypoventilation	NMD-CDEs
% Patients on assisted ventilation	NMD-CDEs
%NIV, %night-only NIV, %exacerbation only NIV	NMD-CDEs
% Patients on invasive ventilation	NMD-CDEs
% Patients with swallowing dificulties	NMD-CDEs
% Gastrostomy, weight variation in one year before gastrostomy	NMD-CDEs
Number of days per year with nasogastric tube feeding	NMD-CDEs
% Patients with cardiac diagnosis/by strata	NMD-CDEs
% Patients with pacemaker (PM)	NMD-CDEs
% Patients with Implantable Cardio Defibrillator (ICD)	NMD-CDEs
% Patients with Cardiac Resynchronization Therapy (CRT)	NMD-CDEs
% Patients with Heart Transplantation (HT)	NMD-CDEs
% Patients according to medication / medication groups	NMD-CDEs
% Patients with scoliosis surgery	NMD-CDEs
Number of unplanned hospitalization per year	NMD-CDEs
Number of days of unplanned hospitalization per year	NMD-CDEs
% Patients with access to physical therapy per year	NMD-CDEs
% Patients with access to occupational therapy per year	NMD-CDEs
Time from first HCP visit to initiation of specific/targeted therapy	NMD-CDEs
% Patients in other registries	NMD-CDEs
% Patients in clinical trials	NMD-CDEs
% Patients on specific treatment designated in 21.2 stratified by dosage	NMD-CDEs
Treatment response stratification	NMD-CDEs
% Patients having received a specific diagnosis after 12 months	NMD-CDEs
% Patients receiving “Mitococktail” or food supplements (yes / no?)	DS-DEs
% Patients with partial / complete remission (CIDP)	DS-DEs
Change of ambulation in the last 12 months	DS-DEs
Time to full remission	DS-DEs
Time to partial remission	DS-DEs
Time from symptom onset to thymectomy	DS-DEs
% Patients with complete remission (CIDP)	DS-DEs
% Patients with partial remission (CIDP)	DS-DEs
% Patients with dysimmune neuropathies with anti-nerve antibodies	DS-DEs
Stratification by antibodies of patients with dysimmune neuropathies	DS-DEs
Stratification by antibodies titer	DS-DEs
% Patients with complete remission after 12 months	DS-DEs
Scale of activities (of daily living) for specified diseases	DS-DEs
% Patients with partial remission after 12 months	DS-DEs
% Patients screened for malignancies	DS-DEs
% Patients with access to speech therapy per year	DS-DEs
% Patients with access to dietary advice per year	DS-DEs
Maximum walking distance estimate	DS-DEs
% Patients QOL (quality of life)	DS-DEs
% Patients discussed in multidisciplinary teams (MDT) / CPMS / boards	DS-DEs
% Patients with mito-associated epilepsy per year	DS-DEs
% Patients with epilepsy treatment following existing guidelines each year	DS-DEs
% Patients receiving "Mitoocktail" or food supplements	DS-DEs
Stratifcation by mitochondrial phenotype	DS-DEs
Stratifiation by score (NMDAS/NMPDS/IPMDS)	DS-DEs
Number of days ventilated per patient per year	DS-DEs
SMN2 copy number	DS-DEs
% Patients with definitive ALS diagnosis	DS-DEs
Stratificaion by ALS presentation form	DS-DEs
Stratificaion by other ALS presentation symptoms or signs	DS-DEs
Stratification by ALS-FRS-R score	DS-DEs
Number of patients evaluated with disease-specific outcome measures	DS-DEs

## Abbreviations

CDEs: Common data elements
DPIA: Data protection impact assessment
DS-CDEs: Disease-specific date elements, only disease or disease-group specific data elements
EC: European Commission
EJPRD: European Joint Programme on Rare Diseases
ERDRI: European Rare Disease Registry Infrastructure
ERNs: European Reference Networks
EU: European Union
EU-CDEs: Joint Research Centre of the European Commission set of common data elements applying to all rare disease registries
FAIRification: Application of the FAIR data principles of data stewardship to one or more data elements
GDPR: General data protection regulation
HTTPS: Hypertext transfer protocol secure (HTTPS) is a protocol for data transmission widely used in the Internet, that uses encryption for secure communication over a computer network
KPIs: Key performance indicators
HCPs: Healthcare providers, that is hospitals or centres of expertise forming the nodes of the ERN
HPO: Human phenotype ontology
NMD: Neuromuscular disorder
NMD-CDEs: Neuromuscular common data elements, consist of all data elements shared by all neuromuscular diseases
Pseudonymization: According to GDPR, it is “the processing of personal data in such a way that the data can no longer be attributed to a specific data subject without the use of additional information, as long as such additional information is kept separately and subject to technical and organisational measures to ensure non-attribution to an identified or identifiable individual”
RD: Rare disease(s)
RDF: Resource description framework
Triple: A semantic triple, RDF triple or simply triple, is the atomic data entity in the resource description framework (RDF) data model. A triple is a set of three entities that codifies a statement about semantic data in the form of subject–predicate–object expressions (e.g., "Jack is tall”)
Triplestore or RDF store: Purpose-built database for the storage and retrieval of triples through semantic queries [4]
